# Platelets and platelet-derived extracellular vesicles: their role in lung cancer dissemination and premetastatic niche formation

**DOI:** 10.3389/fimmu.2025.1703974

**Published:** 2025-11-26

**Authors:** Camilla Locatelli, Nicole Ferrario, Orazio Fortunato, Patrizia Ghidotti, Rossella Crescitelli

**Affiliations:** 1 Epigenomics and Biomarker of Solid Tumors Unit, Fondazione IRCCS Istituto Nazionale dei Tumori, Milan, Italy; 2 Sahlgrenska Center for Cancer Research, Department of Surgery, Institute of Clinical Sciences, Sahlgrenska Academy, University of Gothenburg, Gothenburg, Sweden

**Keywords:** lung cancer, extracellular vesicle (EV), platelet, metastasis, microRNA

## Abstract

Metastasis, the primary cause of cancer-related mortality, is sustained by complex interactions between tumor cells and host-derived factors. Extracellular vesicles, membrane-bound particles that mediate intercellular communication, have emerged as critical regulators of this process. Among them, platelet-derived extracellular vesicles represent the most abundant EV population in circulation and extend the multifaceted influence of platelets in cancer progression. Platelets actively contribute to metastasis by shielding circulating tumor cells from immune surveillance, promoting vascular remodelling, facilitating extravasation, and releasing soluble factors that shape the premetastatic niche. Platelet-derived extracellular vesicles further potentiate these processes by delivering a heterogeneous cargo of proteins, nucleic acids, and lipids to endothelial, stromal, and immune cells, thereby promoting angiogenesis, extracellular matrix remodelling, immune suppression, and organ-specific metastatic colonization. This review summarizes current evidence on the cooperative roles of platelets and platelet-derived extracellular vesicles in metastatic dissemination, with particular emphasis on their contribution to lung premetastatic niche formation and their emerging translational potential in oncology.

## 1 Introduction

Metastasis is the process by which cancer cells spread from their original location to other parts of the body, forming new tumors (1), and it represents one of the major contributors to cancer-related deaths (2). Furthermore, morbidity and mortality of metastasis are associated with the rise of paraneoplastic syndromes and complications due to treatments (2). Metastasis is a highly complex process involving numerous biological components. Identifying its key contributors is essential for the development of metastasis-targeted therapies and for improving the management of advanced disease (3).

Blood represents a complex fluid comprising different cellular (red blood cells, white blood cells, and platelets) and soluble components, many of which are actively involved in the metastatic process (4, 5). Platelets are the most abundant cell population in blood, and their primary activities are related to the coagulation cascade and hemostasis maintenance (6). Among other factors, such as cytokines and growth factors, extracellular vesicles (EVs) are highly present in the blood circulation. EVs are membrane-limited particles released by almost all cells in our body (7). Blood EVs originate primarily from blood cells and endothelial cells, although vesicles released from distant organs can also be detected in circulation (8). Platelets and platelet-derived extracellular vesicles (PEVs) are two major contributors to tumor progression and metastasis establishment, as they are involved in nearly all steps of the metastatic cascade (9). The complex bidirectional interaction between cancer cells and platelets has important clinical implications in metastasis management. This interaction can lead to platelet activation with consequent rise of thrombotic complications that represent a major cause of cancer-related deaths (10, 11). Moreover, cancer cells exploit platelets to enhance their chances of establishing metastases in distant organs and to develop resistance to cytotoxic chemotherapeutic drugs (12–14). This review summarizes the key mechanisms by which platelets contribute to metastatic establishment and how PEVs influence the formation of the premetastatic niche (PMN), with particular emphasis on PMN formation in the lungs.

## 2 Metastatic cascade in solid tumors

The first event in the metastatic cascade is the escape of metastatic cells from the primary tumor, followed by their invasion of nearby lymphatic vessels or the bloodstream. In this phase, metastatic cells acquire a more plastic phenotype by undergoing the epithelial-to-mesenchymal transition (EMT) and acquiring different genomic alterations (15, 16). As a consequence of these events, metastatic cells detach from surrounding cells and extracellular matrix (ECM) and migrate towards the circulation.

Intravasation is a critical bottleneck in the metastatic process. Evidence from mouse models demonstrates that, although large numbers of tumor cells are shed from solid tumors, fewer than 0.1% successfully form metastases at distant sites (17, 18). This low rate of success could be explained by the fact that once in the bloodstream, tumor cells have to face different threats, including as anoikis due to cellular detachment, shear stress, and the presence of immune cells, making the circulation a hostile environment for their survival. In the circulation, circulating tumor cells (CTCs) have been detected as both single cells and as clusters of cells, and their presence in the circulation is generally associated with a worse prognosis for patients (19, 20). Furthermore, CTCs could be circulating in association with other cells, such as neutrophils (20) and platelets, which increases their survival probability and, at the same time, avoids their recognition and consequent elimination mediated by natural killer (NK) cells and other cytotoxic immune cells (21).

As a consequence, CTCs can extravasate into distant organs and initiate metastatic colonization (22). Generally, each primary tumor shows a specific organ tropism for the metastatic seeding. This behavior forms the basis of Paget’s classic ‘seed and soil’ theory of metastasis (23). It is known that the metastatic behavior of tumor cells is affected by cell intrinsic properties but also by a plethora of different environmental cues, such as chemokines, cytokines, and EVs released by both primary tumor cells and the cell components of the host microenvironment. All these factors contribute to the formation of the so called PMN, which favors colonization and organ-specific metastatic dissemination (24).

Once in secondary organs, disseminated tumor cells (DTCs) face new challenges that undermine the establishment of metastasis. At this level, DTCs can be eliminated by patrolling immune cells (25) or nutrients’ deprivation, hypoxia (26, 27), and elevated oxidative stress (28, 29). To overcome these difficulties, DTCs can enter into dormancy, which allows their persistence in secondary organs for months or even decades, thus avoiding elimination by immune cells and chemotherapeutics (30–32). Several factors then mediate the following re-awakening of cancer cells. Integrin signaling and interactions with the ECM have been implicated in the exit from dormancy, enabling metastatic outgrowth. Integrin-β1 signaling is a well-known regulator of this process: its inhibition induces cell cycle arrest. It sustains dormancy in various cancer models (33), whereas its upregulation facilitates re-entry into the cell cycle (34). Cancer cell proliferation at metastatic sites is likewise induced by the interaction between collagen and noncanonical discoidin domain receptor 1 (DDR1) (35). Furthermore, in a model of dormant breast cancer and lung adenocarcinoma, the depletion of the WNT ligand DKK1 promotes the re-entrance into the cell cycle, highlighting an important role of WNT pathway activation for the metastatic progression (36).

### 2.1 Pre-metastatic niche

The mechanisms that establish a favorable and protective microenvironment in secondary organs, facilitating tumor cell colonization and growth before the arrival of CTCs, are referred to as PMN formation (24, 37). The concept of PMN as currently known was first introduced by Lyden and colleagues, who outperformed and ameliorated both Paget’s “seed and soil” theory and Ewing’s assumption (38, 39). Indeed, growing evidence highlighted that organotropic metastases and the development of the PMN are a combination of multiple factors and an intricate interplay among tumor-secreted molecules and microenvironment alterations (40). The PMN is characterized by some main features, including vascular permeability and angiogenesis, lymphangiogenesis, inflammation, immunosuppression, stromal and ECM remodeling, and metabolic reprogramming (41). Each step is finely regulated by an intricate interplay of cells and soluble factors that ensures the establishment of a suitable soil for the attachment and growth of primary tumor cells (42). Angiogenesis and disruption of the endothelial wall are early events in PMN formation, and as the ultimate goal, they enable tumor cell extravasation, thereby facilitating metastasis (43). Mechanistically, vascular leakage is driven by molecules that disrupt the vascular basement membrane and alter tight junctions, such as zonula occludens-1 (ZO-1), occludin and claudin-5, in endothelial cells (44). A plethora of factors released by tumor cells or tumor microenvironment (TME) cell populations are involved in the process of new vessel formation. Kaplan et al. demonstrated that VEGFR1+ bone marrow-derived cells (BMDCs) are essential for creating a suitable environment for secondary tumor attachment in response to primary tumor signals, by promoting angiogenesis in PMN (45). Other cell populations contribute to the neo-angiogenesis process, such as cancer-associated fibroblasts (CAFs) (46, 47). Indeed, they have been shown to release the lncRNA SNHG5, which upregulates CCL2 and CCL5, thereby activating the p38 MAPK signaling pathway in endothelial cells within the PMN. This activation promotes angiogenesis, enhances vascular permeability, and supports the establishment of the premetastatic microenvironment (48). Moreover, macrophages, in addition to their capacity to secrete VEGF, also release CXCL1 and CXCL8, which further promote angiogenesis (49). Hypoxia and its key transcriptional mediators, hypoxia-inducible factors 1α (HIF-1α) and hypoxia-inducible factors 2α (HIF-2α), are critical regulators of blood vessel formation. These factors promote the recruitment of endothelial progenitor cells from the bone marrow and their differentiation into endothelial cells through the regulation of VEGF expression. In addition, HIF-1α and HIF-2α facilitate angiogenic remodeling by inducing the expression of matrix metalloproteinases (MMPs) and lysyl oxidase family enzymes (LOX, LOXL2, and LOXL4), which contribute to the sprouting of pre-existing vessels and the remodeling of collagen fibers within the extracellular matrix (50, 51).

The ECM has been widely recognized as a key factor in the formation of distant pre-metastatic and metastatic niches, with its alteration and remodeling playing a crucial role in shaping the tumor microenvironment and determining the fate of tumor cells (52). The ECM is composed by a complex structure that includes various proteins, namely collagens, elastins, fibronectins, glycoproteins, laminins and ECM-associated proteins (53). By considering the alteration of ECM in relation to its abundance, tumour cells are shown to orchestrate the recruitment of stromal cells that produce various pro-fibrotic growth factors and inflammatory factors such as TGF-α, TGF-β, fibroblast growth factor (FGF)-2, platelet-derived growth factor (PDGF), and epidermal growth factor (EGF) (54). In a pro-tumorigenic context, structural modifications of the ECM play a key role. Recent studies have shown that fibrillar collagen accumulation directly promotes tumor development and progression (51), while fibronectin deposition creates a supportive niche for the adhesion of BMDCs, which are critical for PMN formation (55). Moreover, ECM modification can be mediated by the mechanical force applied by the integrins that modulate the basement membrane conformation, thus facilitating cancer cell invasion (56). Critical enzymes involved in ECM remodeling are MMPs. Namely, MMP9 is over-expressed by endothelial cells and MAC1+/VEGFR1+ myeloid cells in PMN, and its expression has not only been associated with tumor cell invasion but also with the recruitment of BMDC to the niche. Moreover, MMP9, together with MMP2 mediates tumor cells’ invasion through degradation of collagen IV (57, 58).

Besides the ECM context, inflammation and immunosuppression are two critical points, strictly correlated to each other in the PMN formation (41). It has been demonstrated that tumor cells release inflammatory cytokines (IL-6, IL-1, TNF-α), chemokines (CCL2, CCL5, CCL15, CCL26), and growth factors (TGFβ), which ultimately recruit BMDCs such as myeloid-derived suppressor cells (MDSCs) and create a favorable niche for tumor development (59, 60). For example, S100 proteins have been linked to inflammation and the recruitment of hematopoietic progenitor cells and immune suppressor cells, such as regulatory T cells (Tregs), and tumor-associated macrophages, which help tumors evade immune detection (61). Similarly, macrophages resident in premetastatic sites were shown to contribute to the establishment of an immunosuppressive microenvironment with inflammatory characteristics (62). Furthermore, deregulated TLR4 signaling in tumor cells can lead to an inflammatory response, which potentiates tumor cells’ resistance towards cell death, proliferation, invasion, and metastasis (63). All the processes involved in the PMN cascade are summarized in **Table 1**.

**Table 1:** Key stages, mechanisms, and molecular players involved in PMN formation.

PMN stage	Mechanisms	Molecules involved	Ref
Vascular leakness	Alteration of vascular basement membrane and tight junctions	ZO-1, claudin-5	(44)
Angiogenesis	Promotion by BMDCs	VEGFR1	(45)
	Stimulation of P38MAPK signaling pathway in endothelial cells by CAFs	CCL2, CCL5	(48)
	Promotion by macrophages	VEGF, CXCL1, CXCL8	(49)
	Hypoxia-induced recruitment of EC progenitor cells from bone marrow	HIF-1α, HIF-2α,	(64)
	Promotion of vessel sprouting by hypoxia	MMPs, LOX, LOXL2, LOXL4	(51)
Stromal and ECM remodeling	Production of pro-fibrotic and inflammatory factors by stromal cells	TGF-α, TGF-β, FGF-2, PDGF, EGF	(52)
	Accumulation of ECM	Fibrillar collagen, fibronectin	(53)
	Alteration of basement membrane conformation	Integrins	(54)
	ECM alteration	MMP9, MMP2	(57)
Inflammation/immunosuppression	Recruitment of BMDC	MMP9, MMP2	(58)
	Recruitment of MDSC	IL-6, IL-1, TNF-α, CCL2, CCL5, CCL15, CCL26, TGFβ	(59, 60)
	Induction of an inflammatory microenvironment and recruitment of anti-tumor cells	S100 proteins	(61)
	TLR4 pathway dysregulation		(63)

### 2.2 Lung metastasis formation

The lung represents a preferential organ for metastasis formation for many types of tumors due to its high vascularization, ECM composition and organization (65). In primary lung cancer, intrapulmonary metastases, either contralateral or ipsilateral, are observed in approximately 15–30% of cases (66). Moreover, in breast cancer, the lung is a frequent site of metastasis, with an incidence of about 40% in triple-negative and 20% in non-triple-negative subtypes (67). Lung metastases are also frequent in several other malignancies, occurring in approximately 46% of prostate cancers, 40–50% of renal cell carcinomas, 17–40% of sarcomas, and about 6% of hepatocellular carcinomas (66). Hematogenous dissemination to the lungs represents the most common metastatic route for many cancer types, given the continuous inflow of blood from the heart (66). Through the bloodstream, CTCs, tumor-derived extracellular vesicles (TEVs), and other soluble factors can reach the lungs, where they modulate the local microenvironment and promote PMN formation and subsequent metastatic colonization (68). Reactivation of disseminated tumor cells is promoted by remodeling of the ECM, transitioning from a disorganized to a highly aligned configuration rich in type III collagen fibers. This structural change triggers cancer cell proliferation through activation of the DDR1–STAT1 signaling axis (69). In addition to their role in ECM remodeling, lung-resident fibroblasts are essential for establishing an immunosuppressive microenvironment that facilitates metastatic growth (70). In particular, by producing prostaglandin E2, lung fibroblasts impair dendritic cell (DC) function and promote the expansion of suppressive monocytes (70). The spatial distribution of immune cells also contributes to the development of lung metastases (66). In breast cancer metastasizing to the lungs, metastatic regions have been shown to be enriched in macrophages, macrophage-regulatory cells, and monocytes producing type I interferons (IFNs) (71). In contrast, anti-tumor immune populations, such as T cells and NK cells, are primarily detected in lung regions devoid of metastases. This spatial organization supports metastatic outgrowth, with tumor-promoting immune cells concentrated within the metastatic core and anti-tumor immune cells excluded to the periphery. The recruitment and functional hijacking of immune cells further contribute to neo-angiogenesis within the metastatic niche (71). Tenascin C, produced by cancer cells, stimulates lung-associated macrophages via TLR4 to produce nitric oxide and TNF, thereby inducing an inflammatory response in endothelial cells and supporting the formation of a metastatic vascular niche (72). In an inflammatory context, endothelial cells are also responsible for the proliferation of cancer cells through the production of TGF-β1 and periostin (73). Furthermore, in breast and prostate cancer lung metastasis models, inflammation has been shown to trigger neutrophils to release extracellular traps, leading to laminin degradation and the generation of signals that awaken dormant cancer cells (74).

## 3 Extracellular vesicles and cancer

EVs are defined as membrane-limited particles released by all kinds of cells and cannot replicate on their own (75). Initially identified as cell waste, these particles have recently gained increasing attention for their fundamental role in intercellular communication, both in physiological and pathological conditions (76). “Extracellular vesicles” is an umbrella term that encompasses different particle subtypes that differ based on their biogenesis, size, and composition. In this review, we will use the term EVs consistently, irrespective of the terminology adopted by the cited authors.

The biological functions of EVs largely stem from their capacity to influence recipient cells by delivering bioactive molecules such as proteins, lipids, and nucleic acids either as cargo or displayed on their surface (77). EVs are released in different biofluids, allowing them to reach distant organs. Interactions with target cells occurring through specific receptors or adhesion molecules directly modulate the cell’s behaviour and function (78, 79). However, the effector functions of EVs are often exerted after internalization into cells (80). Once internalized, the EV cargo is released and can modulate multiple signaling pathways within the target cell (77).

In recent years, growing evidence has highlighted the role of EVs in nearly all stages of tumor development and progression (81). TEVs modulate the behaviour of stromal and immune cells within the microenvironment, reprogramming them to support cancer growth. They also play a pivotal role in PMN formation, underscoring their capacity to influence distant organs (82). EVs could contribute to the local and systemic cancer progression by enhancing the proliferation and survival of tumor cells as they can carry a wide range of bioactive molecules, including proteins, DNA, RNA, lipids, and metabolites (83). EV cargo includes oncogenic proteins (e.g., EGFR, c-Myc) and regulatory RNAs that enhance cancer cells’ proliferation, cell invasiveness, and metastatic potential by inducing EMT, promoting ECM degradation, and conditioning distant tissues through the establishment of PMN (84, 85). Moreover, EVs play a prominent role in angiogenesis, facilitating tumour vascularisation through the delivery of pro-angiogenic factors as VEGF, FGF, and angiogenesis-related miRNAs (86). EVs also facilitate immune evasion by impairing the function of cytotoxic immune cells and reprogramming immune responses to promote tumor tolerance (83) (71, 87).

Beyond their autocrine effects, TEVs are especially effective at corrupting cells within the tumor microenvironment (TME) to promote cancer progression. For example, lung cancer–derived EVs boost angiogenesis and vascular permeability by delivering miR-23a to endothelial cells, which causes HIF-1α accumulation and the breakdown of the tight junction protein ZO-1 (88). Fibroblasts are another common target of TEVs, often reprogrammed into CAFs. In both prostate and bladder cancer, TEV-associated TGF-β1 induces stromal fibroblasts to acquire a myofibroblast-like phenotype by activating the SMAD signaling pathway, thereby promoting tumor growth and angiogenesis *in vivo* (89, 90).

Another crucial aspect of TEVs is their immunomodulatory properties (91). TEVs can promote immune evasion by shutting down NK cell and cytotoxic T cell functions, as well as activating pro-tumoral immune cells, such as MDSCs (92), TAMs (93, 94), and Tregs (95).

TEVs also exhibit tumor-associated antigens (TAAs) and damage-associated molecular patterns (DAMPs) on their surface. They have immunogenic properties exerted on antigen-presenting cells (DCs) and can trigger an anti-tumor immune response (91). Collectively, TEVs have multifaceted roles in cancer biology, orchestrating interaction between tumor and distant organs, reshaping the TME, and modulating the immune system.

## 4 Platelets

In the 19^th^ century, Giulio Bizzozero identified a third morphological cell population within the blood, separate from white and red blood cells: platelets (96). He described platelets as anucleate, disc-shaped structures, round or oval, about three times smaller than erythrocytes. Today, we know that platelets are approximately 2-4 μm in size and result from the fragmentation of megakaryocytes (MKs) (97). MKs, large multinucleated cells found in the bone marrow, spleen, and lungs, shed platelets into the bloodstream, where they have a lifespan of approximately 5–7 days (98, 99). Platelets inherit granules, mitochondria, coding and non-coding RNAs from MKs, as well as translational machinery for post-transcriptional gene regulation (100). They are primarily involved in haemostasis and coagulation processes, thanks to the release of several factors (101, 102). Platelets contain several critical structures, including α-granules, dense granules, and lysosomal granules, all of which are essential for optimal function. These proteins are released upon activation and are derived from the continuous endocytosis process of MKs and platelets (103).

### 4.1 Platelets’ physiological functions

Platelets play vital roles in maintaining vascular integrity and tissue homeostasis. Their classical function is the clot formation (101). Platelet-dependent coagulation can be activated through two mechanisms, which imply the activation of two distinct pathways: the extrinsic and the intrinsic pathways. The extrinsic pathway begins with endothelial injury, which triggers the release of tissue factor (factor III) into the blood, leading to its processing and activation of the typical cascade (104, 105). The intrinsic pathway begins, instead, when factor XII, also known as the Hageman factor, is activated by exposure to collagen, kallikrein, and high-molecular-weight kininogen (HMWK) (106). In this case, the cascade proceeds through the activation of factors XI and IX before merging into the common coagulation pathway shared by both routes (106, 107). The final steps of the coagulation cascade aim to produce thrombin and fibrin, creating a solid structure that prevents further bleeding (107).

Platelets preserve the vascular integrity by releasing sphingosine-1-phosphate (S1P), a bioactive lipid that protects the endothelial barrier and prevents leakages (108). They are also recognised for their involvement in other processes, including inflammation, where they recruit leukocytes to the damage site, angiogenesis, and tissue regeneration (109, 110). All these mechanisms are regulated by the release of growth factors, cytokines, and EVs by platelets that modulate the revascularisation and healing of connective tissue damage (111).

### 4.2 Platelets in cancer

**Figure 1:**
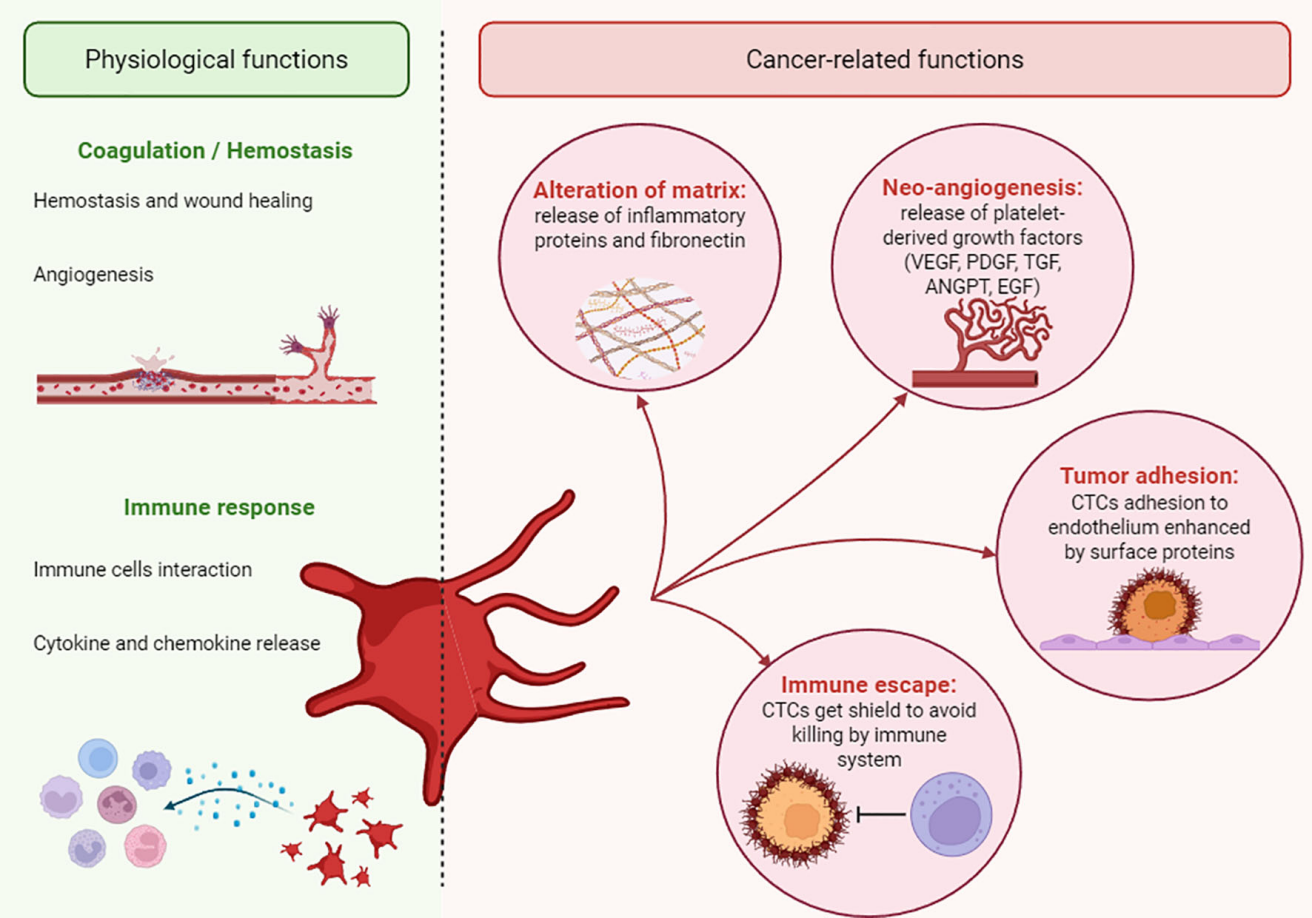
Physio-pathological roles of platelets. A schematic illustration of the dual role of platelets. The pathological roles of platelets in cancer are indicated as “cancer-related functions”.

The primary role of platelets is to maintain blood homeostasis, but they can also participate in pathological processes. Several studies show an association between platelets and the onset and progression of cancer (**Figure 1**). For example, platelets are responsible for the tumour’s immune evasion, tumour cell adhesion and arrest on the endothelial wall, as well as their extravasation and survival (112). Different mechanisms, dependent on the environment, are used by platelets to support tumour growth (95).

In the bloodstream, platelets help CTCs released by melanoma, breast, and lung cancer to survive in the circulation by preventing immune cell binding and killing (113). Specifically, fibrinogen and tissue factor, along with NKG2D downregulation, help platelets form a shield over CTCs to prevent tumor cell recognition by NK cells (114). Additionally, the presence of selectins and integrins on the surface of platelets is crucial for the arrest and adhesion of CTCs on the endothelium (115).

Within the TME, platelets release proangiogenic factors, including interleukins, VEGF, CXCL12, and TGFβ, which promote neovascularisation, EMT, and metastatic seeding (116, 117). One example is platelets’ ability to induce the biosynthesis and esterification of 12S-hydroxyeicosatetraenoic acid (12S-HETE) in colon cancer cells, which, in turn, modulates the expression of EMT marker genes and promotes metastasis (118). Although these findings are important for the development of new therapeutic strategies, free 12S-HETE levels were not significantly different between colorectal polyps, cancer mucosa, and normal colorectal mucosa in humans. Furthermore, as the cited study was conducted exclusively in cell lines, it would be valuable to validate these results in animal models and human samples (118). Other mechanisms, such as coagulation-associated pathways and fibrinogen production, are upregulated in lung adenocarcinoma compared to squamous cell carcinoma, suggesting that platelets play a significant role in advanced and metastatic stages (119).

### 4.3 Platelets’ activities during tumor growth

Beyond releasing bioactive molecules, platelets also actively uptake factors from the bloodstream, thereby changing their cargo composition and undergoing a process of “education” (95). These factors may originate from the bone marrow, stromal cells, or cancer cells, influencing platelet functions and promoting a pro-tumorigenic phenotype (120). Platelets that undergo this process, known as tumor-educated platelets (TEPs), acquire a unique RNA cargo capable of distinguishing between cancer patients and those with non-malignant or inflammatory conditions (121). The RNA in TEPs undergoes extensive splicing, resulting in an enrichment of mRNAs related to vesicle transport and cytoskeletal functions, as well as miRNAs that regulate gene expression and RNA silencing in recipient cells. These findings are based on a detailed analysis of platelet RNA from healthy donors and patients with localized or metastatic cancer (121). The authors showed that sequencing could accurately identify the diagnosis and location of the primary tumor, offering hope for the future of platelet-based liquid biopsies (121). Although these results are promising, the study emphasizes that inflammatory diseases and other factors, such as cardiovascular events and non-cancerous conditions, may also influence the platelet mRNA profile. A key point in the study is the selection of the healthy control group, which consists of younger individuals than those in the cancer group. Since the RNA content of platelets varies with age and gender (122), some of the observed differences may be attributed mainly to the age gap between the two groups.

Additionally, *in vitro* studies in which platelets were co-cultured with cancer cells or exposed to conditioned media from cancer cell cultures demonstrated enhanced platelet activation and alterations in platelets RNA signatures (123). These interactions also promoted cancer cell survival, migration, and invasion by activating the TGFβ/Smad/PAI-1 and PI3K/AKT signalling pathways (124, 125). These findings are based on indirect interactions between platelets and CTCs, suggesting that the factors responsible for platelet education are present in the conditioned medium. In circulation, platelets could promote PMN formation and metastasis by directly interacting with CTCs (126). Recent evidence further reveals that platelets can physically cover CTCs in the bloodstream, shielding them from the immune system (127, 128).

*In vivo*, platelets can extravasate from the bloodstream and infiltrate the tumor stroma, where they are identified as tumor-infiltrating platelets (TIPs) through histochemical detection of CD42b expression. TIPs accumulation is elevated within tumor tissue and correlates with cancer progression and stage across different tumor types (129). The presence of TIPs could thus be used to integrate the RNM staging system as well as predict the prognosis and post-surgical survival in several cancer types, including pancreatic ductal adenocarcinoma and colorectal cancer (130, 131).

Elucidating the role of TEPs and their oncogenic cargo has significant implications for cancer diagnosis and therapy. TEPs represent promising biomarkers of cancer progression and potential targets for strategies aimed at blocking their pro-tumorigenic activities (132). Notably, key mRNA markers such as MAX, MTURN, UQCRH, and HLA-B are significantly upregulated in TEPs and have been associated with chemotherapy responses, underscoring their value as non-invasive biomarkers for cancer detection (133). Additionally, TEPs and TIPs can modulate the TME and its vascular supply by releasing factors and EVs enriched in regulatory miRNAs (132, 134).

#### 4.3.1 PEV cargo in tumor progression

EVs carry a complex cargo that mirrors the state of their cells of origin (75). Among them, PEVs are the most abundant population in the bloodstream, generated through the continuous release of vesicles that occur during normal platelet physiology (135). PEVs inherit both cytosolic and membrane components from platelets and are characterized by specific membrane markers, including CD41, CD42a, CD42b, and CD62P (136, 137). Their concentration is closely linked to the platelet activation state, which can be altered under pathological conditions (137). In cancer, platelets and PEVs act as potent immunomodulators, exerting both suppressive and stimulatory effects. They shield CTCs, transfer PD-L1 and TGF-β, and release prostaglandin E_2_, collectively inhibiting CD4^+^ and CD8^+^ T cell activity and promoting an immunosuppressive TME (138, 139). Moreover, PEVs acquire diverse tumor-derived biomolecules, including proteins (such as cytokines and enzymes), nucleic acids (coding and non-coding RNAs), second messengers, and even mitochondrial components (140, 141). This heterogeneous cargo underpins the diverse biological activities of PEVs across different target cell types. Importantly, EVs can cross the bloodstream and tissue barriers, enabling PEVs to deliver their cargo to distant cells and organs (142). Through this mechanism, PEVs mediate communication between the tumor and the microenvironment, thereby fostering growth, metastasis, and overall cancer progression.

#### 4.3.2 MicroRNAs

PEVs play a crucial role in mediating intercellular communication by transferring microRNAs between cells. MiRNAs are small nucleic acids, ranging from 19 to 25 nucleotides in length, that play an essential role in regulating RNA expression. Their presence in EVs significantly affects various types of cancers and the pathophysiology of the immune system (142). These vesicles can be internalized by recipient cells, reshaping their molecular profiles and functions (143). Specific miRNAs carried within PEVs promote cancer aggressiveness by driving invasion, migration, and angiogenesis (143). For example, it has been shown that miR-939 found in PEVs is delivered to ovarian cancer cells and is linked to increased aggressiveness (144). However, in the cited study, healthy donor platelets stimulated with thrombin were used as controls. This condition does not accurately mirror the physiological state of cancer patients and thus limits clinical relevance. Moreover, PEVs transporting miR-223 are internalized by endothelial cells, where they downregulate the tumor suppressors FBXW7 and EFNA1 (145). This study suggests that PEVs can be internalized by diverse cell types beyond tumor cells. Specific miRNAs released by PEVs, including miR-126, let-7a, and miR-320b, are implicated in the regulation of angiogenesis. Notably, miR-126 promotes angiogenesis and modulates CXCL12 and VCAM-1 expression in endothelial cells, thereby facilitating transendothelial migration and contributing to vascular inflammation (146, 147). However, as the vesicles analysed were generated through *in vitro* platelet lysis, it remains unclear whether they accurately reflect EVs naturally secreted by circulating platelets, limiting insight into their proper pathophysiological role. Given the central role of miR-126 in multiple angiogenic pathways, further studies are necessary to elucidate these mechanisms and establish their biological significance. PEV-mediated delivery of let-7a to endothelial cells provides an angiogenic stimulus that supports the growth of solid tumors (148). However, in this study, EVs were derived from healthy donor platelets activated with thrombin, a condition that does not fully reflect the pathological state of cancer patients.

In contrast, a different study successfully isolated plasma-derived EVs from lung cancer patients. It demonstrated the transfer of miR-320 to human umbilical vein endothelial cells (HUVECs), resulting in altered endothelial phenotype (149). More recently, studies have specifically examined miR-320b within PEVs, revealing its role in downregulating ICAM-1 expression in HUVECs (150, 151).

These studies underscore the interplay between TEPs, PEVs, and endothelial cells, which is central to the platelet-mediated regulation of tumor angiogenesis and the TME. Overall, the dynamic activity of EV-associated miRNAs shapes the tumor milieu while also modulating gene expression across diverse cell types.

#### 4.3.3 Additional PEV cargo molecules

It is known that cancer condition alters the composition of proteins in TEP-derived EVs (95). Comparative studies have revealed considerable differences in protein expression between EVs isolated from platelets of colorectal cancer patients and healthy donors. Specifically, the authors identified 119 proteins downregulated and 89 proteins upregulated in EVs from cancer patients compared with those from healthy individuals (152). In the mentioned study, EVs were isolated from the platelets of cancer patients, thereby reflecting the pathological state of the disease. However, in this study, platelets were artificially activated with thrombin to boost EV production, a condition that may alter their composition. Functional assays demonstrated that TEP-derived EVs upregulated key EMT markers in cancer cells, including TWIST and VIM (152). In addition, both colorectal and prostate cancer cells were shown to internalize PEVs, leading to increased expression of MMPs such as MMP-2 and MMP-9, thereby enhancing their ability to remodel the distant microenvironment (153, 154). PEVs also transfer platelet-derived integrins, including CD41, to the surface of tumor cells, which strengthens the adhesion of lung, prostate, and colorectal cancer cells to the endothelium and facilitates their migration and systemic dissemination (155).

Taken together, these findings illustrate the complex interplay between TEP-derived EVs and tumor cells, underscoring their pivotal role in cancer progression and metastasis. Deciphering these mechanisms holds promise for the development of novel targeted therapies and anti-metastatic strategies.

### 4.4 Platelets’ activities during metastatization

During cancer metastasis, TEPs play a pivotal role in PMN formation as illustrated in **Table 2** (167). One key mechanism is tumor cell–induced platelet aggregation (TCIPA), in which platelets encapsulate CTCs, shielding them from immune surveillance and promoting metastatic spread via microthrombus formation (163). TCIPA also fosters lung metastasis in malignant melanoma models through the release of chemokines (CCL2, CXCL12, IL-1α, IL-1β) and recruitment of TAMs, driving their polarization toward an M2 phenotype (168).

**Table 2:** Key stages, mechanisms, and molecules involved in platelet-PMN formation.

PMN stage	Mechanisms	Molecules involved	Ref
Vascular leakness	- Increased endothelial permeability	VEGF, Angpt2, Angptl4, CCL2, fibrinogen, and CXCL12	(156)
		CLEC-2/Podoplanin interaction	(157)
	- Enhanced CTC adhesion and docking on the endothelium	Tissue factor-mediated clot formation	(158)
Angiogenesis	- Recruitment of bone marrow-derived endothelial cells	Secretion of VEGF, vWF, PDGF, TGF-β, and ANGPT-1	(159, 160)
ECM remodelling	- Activation of the MAPK-p42/44 and AKT and upregulation of MMPs	Transfer of CD41 from PEVs to tumor cells	(155)
Inflammation and immune response	- Suppression of IFNγ-mediated responses	BMDC, MDSC and activation of GITR on NK cells	(161)
	- Secretion of pro-inflammatory cytokines	IL-6, IL-10, TGF-β	(161)
	- Formation of neutrophil extracellular traps and macrophages NETosis	PSGL-1/P-selectin interaction	(162)
	- Immune escape	TCIPA and microthrombi formation	(163)
		MHC-I transfer from platelets to tumor cells	(164)
	- Neutrophil activation	GPIb–Mac-1 interactions	(165)
	- NK dampening	miR-183 transferred by PEVs	(166)

At the vascular level, platelets increase endothelial permeability and vascular leakiness, facilitating CTCs extravasation through the secretion of junction-regulating molecules, including VEGF, Angpt2, Angptl4, CCL2, fibrinogen, and CXCL12 (156). They also enhance CTCs adhesion and docking on the endothelium via tissue factor-mediated clot formation, which recruits macrophages expressing CD11b, CD68, F4/80, and CX3CR1 (158). Platelet activation additionally drives granulocyte recruitment (CD11b^+^MMP9^+^Ly6G^+^) through the release of CXCL5 and CXCL7, which bind granulocyte CXCR2 (169, 170). Importantly, granulocyte depletion alone is insufficient to block the formation of metastatic foci, underlining the indispensable contribution of platelets in this early step (169).

Another central pathway involves platelet C-type lectin-like receptor 2 (CLEC-2), which binds podoplanin on tumor cells. This interaction is crucial for vascular permeability changes and cancer cell dissemination: podoplanin-positive lung tumor cells fail to metastasize in Clec2-deficient mice, and CLEC-2 depletion abrogates pro-metastatic thrombus formation *in vivo* (157, 171).

Platelets also contribute to angiogenesis, another key feature of PMN establishment (127). Upon tumor-induced activation, they serve as major transporters of VEGF (159), which triggers the release of von Willebrand factor (vWF) and subsequent secretion of PDGF, TGF-β, and angiopoietin-1 (ANGPT-1). These mediators recruit bone marrow–derived endothelial progenitor cells that drive neovascularization (160).

Beyond angiogenesis, platelets remodel the TME and modulate the immune response. They recruit BMDCs and MDSCs, which suppress IFNγ-mediated responses through Glucocorticoid-induced Tumor Necrosis Factor Receptor Ligand (GITRL) and secrete pro-inflammatory cytokines (IL-6, IL-10, and TGF-β) that enhance inflammation and metastasis (161). Platelets also activate neutrophils to produce neutrophil extracellular traps (NETs) and subsequently undergo NETosis through TLR4 signaling in a P-selectin–dependent manner, thereby promoting platelet aggregation and endothelial activation (172). Activated platelet P-selectin further mediates the recruitment of immune cells (TAMs, monocytes, neutrophils) through binding to PSGL-1 (162). Interestingly, platelets protect tumor cells from immunosurveillance through direct or indirect inhibition of immune cell engagement (173). In breast cancer cell lines, the interaction between platelets and cancer stem cells induced platelet release of TGF-β, which inhibited NK cell activity (174). Additionally, platelet-derived MHC-I can be transferred to tumor cells, shielding them from NK cell–mediated killing and thereby promoting tumor growth (164).

Collectively, platelets are central orchestrators of premetastatic and metastatic niche formation, regulating angiogenesis, immune evasion, vascular remodeling, and ECM dynamics. While many pathways have been elucidated, further studies are required to fully define their mechanistic contributions and therapeutic potential in metastasis.

#### 4.4.1 Platelet-derived EVs and their cargo in cancer metastasis

**Figure 2:**
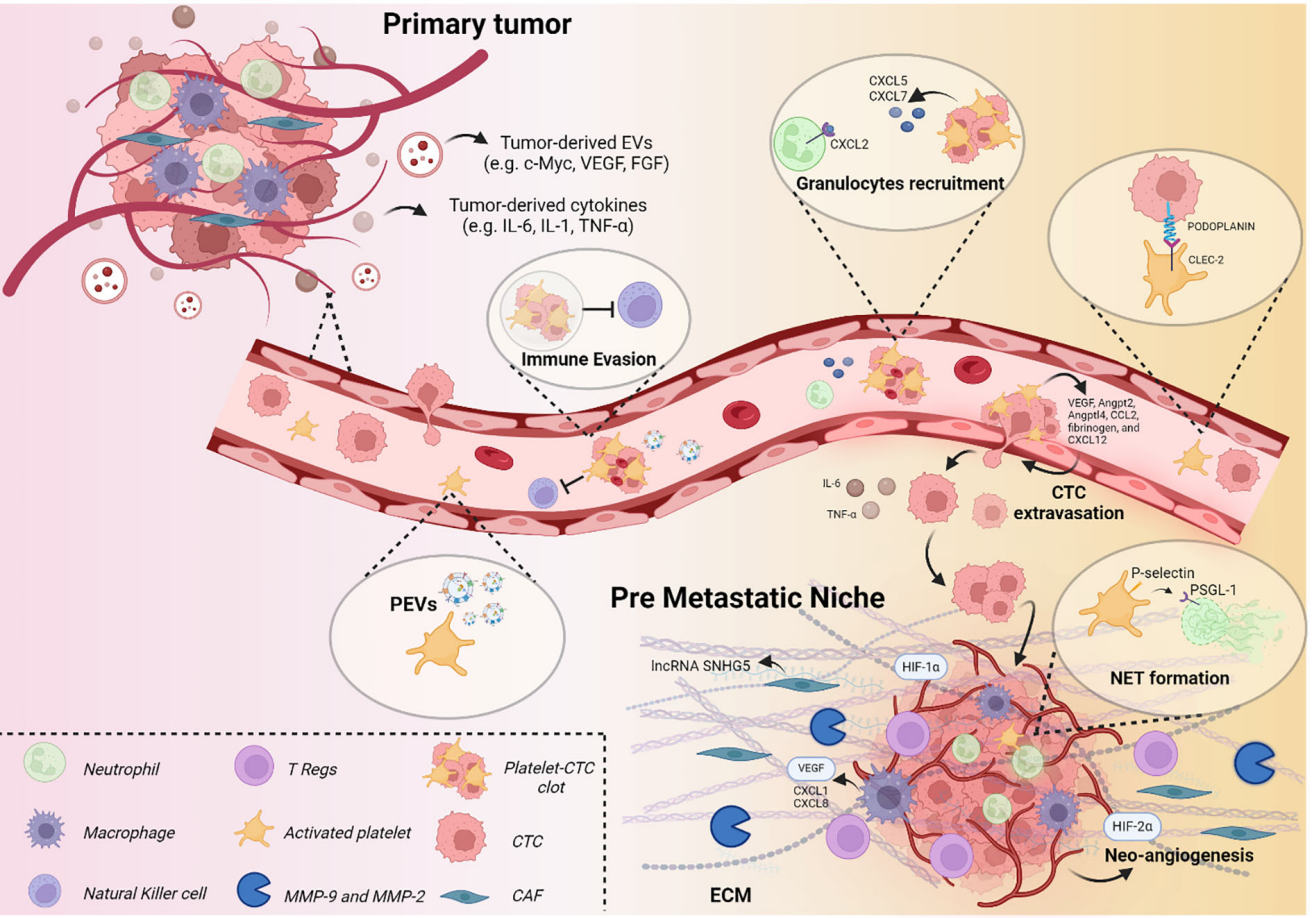
The role of platelets in PMN formation. Schematic representation of soluble factors and cellular composition contributing to PMN establishment, with particular focus on platelet involvement.

Several studies have attributed a critical role to PEVs in regulating various tumor hallmarks, including proliferation, resistance to cell death, invasion, metabolic reprogramming, immunity, and angiogenesis (**Table 3**) (180) (**Figure 2**). Recent studies have demonstrated that the transfer of CD41 from PEVs to tumor cells stimulates the activation of the MAPK-p42/44 and AKT signalling pathways, as well as the upregulation of MMPs necessary for ECM remodelling and invasion (155). PEVs have been shown to enhance tumor cell invasion by stimulating MMP-2 synthesis and secretion, as well as by promoting the transcription of MMP-9, VEGF, IL-8, and HGF mRNAs, factors closely associated with lung cancer metastasis (181). Furthermore, PEVs can transfer miR-223 to different target cells, supporting the metastatic progression (182). In particular, miR-223 promotes invasion and influences the expression of tumour suppressors, such as EPB41L3, in lung cancer cells, thus enhancing their metastatic capability (165). Critical immunoregulatory functions within the TME are achieved by activating neutrophils via GPIb–Mac-1 interactions and by promoting macrophage polarization toward an M2 phenotype (183). Furthermore, PEVs also exhibited the capacity to transfer miR-183 to NK cells, dampening their ability to kill cancer cells (184). Moreover, the transfer of CXCR4 by PEVs, a chemokine receptor that activates key signalling pathways, enhances tumor cell migration and supports the survival of CTCs in circulation (166).

**Table 3:** Summary of clinical studies analysing PEVs in cancer using patient-derived samples.

Cancer type	EV source	Sample size	EV isolation method	Platform	EV characterization	Ref
PDAC	serum	39 patients	MagCapture™ Exosome Isolation Kit Phosphatidylserine	ELISA	NTA, TEM	(175)
Lung	plasma	136 patients	Centrifugation	Flow cytometry	–	(176)
Lung	Platelet-poor-plasma	86 patients	Ultracentrifugation	Flow cytometry	TEM	(177)
Lung	Platelet-poor-plasma	50 patients	Ultracentrifugation	Flow cytometry	–	(178)
Lung	plasma	182 patients	SEC	Flow cytometry	NTA, TEM, WB	(179)

Besides the release of their cargo, the number of PEVs themselves has been shown to play a crucial role in determining the pro-tumorigenic effect (142). PEV concentrations in the blood of lung cancer patients are elevated compared with those of healthy controls. In a cohort of 136 NSCLC patients, PEV levels measured after three months of chemotherapy or targeted therapy were significantly higher in those with disease progression than in patients with controlled disease (176). In addition, Odaka et al. reported that serum levels of PEVs (CD41^+^-EVs and CD61^+^-EVs) were significantly higher in patients with pancreatic ductal adenocarcinoma (PDAC) than in healthy controls, supporting their potential as predictive biomarkers for PDAC (175). *In vitro* and *in vivo* studies by Zhao and colleagues demonstrated that the PKCα agonist PMA increased PKCα levels and enhanced PEV production, thereby promoting lung metastasis in nude mice. Conversely, treatment with the PKCα inhibitor GÖ6976 produced the opposite effect, highlighting PKCα as a key regulator of PEV release (185).

Collectively, these findings point out the pivotal role of platelets and PEVs in metastasis. Deeper investigation into their functions could inform the development of therapies aimed at disrupting platelet–tumor cell interactions. Moreover, given their small size and capacity to mediate intercellular communication, PEVs hold great promise as novel biomarkers and therapeutic targets in oncology.

## 5 Conclusions and future directions

Platelets and their EVs play a pivotal role in cancer progression and metastasis by modulating the TME, shaping immune responses, and promoting PMN formation.

Despite the growing body of literature highlighting the importance of platelets and their EVs in cancer progression, the lack of standardized procedures for EV isolation, purification, and characterization remains a major limitation that hampers cross-study comparability.

First, the heterogeneity of platelet activation stimuli, including thrombin, collagen, lipopolysaccharide, and calcium ionophore, affects not only the number and size of PEVs but also, more importantly, their molecular cargo. This variability complicates the interpretation of findings related to cancer metastasis. Moreover, the absence of universal reference materials and standardized protocols further undermines the accuracy and reliability of quantitative analyses.

A major shortcoming of many published studies is the lack of comprehensive *in vivo* validation, including dose–response assessments, biodistribution analyses, and half-life determinations of PEVs, which are essential to confirm their translational relevance. Indeed, most of the studies discussed in this review were conducted using EVs derived from cell lines or *in vitro* models, without clear *in vivo* confirmation of their functional importance in cancer progression.

To fully harness the potential of platelets and their EVs in biomedical research and therapeutic applications, these methodological and translational challenges must be addressed.

### 5.1 Future directions

To date, no clinical trials are underway to investigate P-EVs in cancer as diagnostic biomarkers or to explore new therapeutic strategies aimed at blocking PEV pro-tumorigenic effects (186). However, several ongoing clinical trials involving platelets focus on anti-aggregating drugs designed to prevent platelet activation, but their results remain inconclusive. For instance, randomized studies assessing aspirin for colorectal adenoma prevention (187–190) demonstrated some preventive effects, although the outcomes were not universally consistent. Likewise, some primary prevention studies have reported inconsistent results; for example, extended follow-up analyses showed that aspirin use was associated with a modest reduction in colorectal cancer incidence and mortality after twenty years (191). Because platelet activation is closely linked to EV release, it is reasonable to hypothesize that anti-aggregating drugs might also inhibit EV secretion into circulation. Further experimental validation of this mechanism could reveal new therapeutic applications for these agents as neoadjuvant treatments in oncology.

As discussed in this review, platelets hold significant potential as biomarkers for the detection and monitoring of various cancer types. However, several technical and methodological challenges, including isolation procedures, stability, storage, and detection, must be addressed before the use of platelets can be fully integrated into clinical practice. Future studies should aim to modulate platelet–tumor interactions to determine whether this approach can meaningfully influence cancer progression and serve as an effective therapeutic strategy, either alone or in combination with standard treatments. Moreover, well-designed clinical trials are essential to establish the safety, efficacy, and translational value of platelet-based strategies in oncology.
